# Adapting and applying intervention mapping to integrate medical-legal partnership into organizations providing HIV care: an implementation methodology study

**DOI:** 10.3389/frhs.2025.1435663

**Published:** 2025-05-01

**Authors:** Miguel Muñoz-Laboy, Ashley French, Robin Davison, Samantha J. Morton, Emily A. Arnold, Abby E. Rudolph, Resa M. Jones, M. Isabel Fernandez, Omar Martinez

**Affiliations:** ^1^ School of Social Welfare, Stony Brook University, Stony Brook, NY, United States; ^2^ College of Medicine, University of Central Florida, Orlando, FL, United States; ^3^ Samantha Morton Consulting, Newton, MA, United States; ^4^ University of California San Francisco, San Francisco, CA, United States; ^5^ College of Public Health, Temple University, Philadelphia, PA, United States; ^6^ College of Osteopathic Medicine, Nova Southeastern University, Fort Lauderdale, FL, United States

**Keywords:** medical-legal partnership, legal services, health-harming legal needs, HIV, people with HIV, implementation science, intervention mapping, health services research

## Abstract

**Background:**

Implementation science in public health has facilitated the translation of research findings into effective public health programming and evidence-based policy decision-making. One of the most prominent implementation science methodologies is intervention mapping, briefly defined as a rigorous protocol that guides the design of multi-level health promotion interventions and implementation strategies. In this manuscript, we describe our use and adaption of intervention mapping in Medical-Legal Partnerships, which are an integration of comprehensive legal services within primary health care and social service spaces working to mitigate the effects of negative social determinants of health for persons with HIV (PWH).

**Methods:**

Intervention mapping in this study was modified from a six-step to a five-step approach by integrating Step 4 and Step 5 of the original version of intervention mapping. The rationale for combining Step 4 and 5 into one step was that coherent, independent intervention packages existed for the provision of legal and HIV services, and it was determined through Step 1 of intervention mapping that these two existing intervention approaches can be integrated at the organizational level but should remain collocated at the patient level. Thus, our modified intervention mapping steps consisted of: (1) conducted needs assessments among medical legal partnerships (MLP) programs (providers and patients) serving PWH in order to identify the current landscape of MLP adoption and implementation in HIV care contexts and common components of those programs; (2) generated organizational and practice-level implementation outcomes and objectives, determinants and change objectives matrices to guide each strategy; (3) chose methods and mechanisms of change of the overarching implementation strategy; (4) produced implementation protocols and materials; and (5) developed a plan to evaluate implementation outcomes.

**Results:**

Following intervention mapping author recommendations that not every step is needed in intervention mapping, using our modified intervention mapping approach resulted in a comprehensive organizational level intervention. Applying our adapted intervention mapping process resulted in the intervention package, OPAHL (Organizational Partnerships for Healthy Living), which currently is being tested for feasibility and preliminary effectiveness in a hybrid, randomized cluster trial in Philadelphia, Pennsylvania.

**Conclusions:**

The work presented here provides a practical framework that can be replicated by other researchers and practitioners working on the social epidemiology of chronic illness, communicable disease, and access to and engagement with care.

## Introduction

Intervention mapping is a six-step approach that specifies processes for integrating empirical evidence and contextual-practical considerations within health intervention planning (1, 2). The standard intervention mapping approach has been demonstrated to be efficient in developing evidence-based interventions in areas of chronic illness, communicable disease, and behavioral change interventions (1–8). Implementation science analyses of intervention mapping suggest that, as a methodology, it increases the cultural relevance of intervention tools, proper adaptability, and effective target population uptake of interventions. Despite the multilevel features of intervention mapping, it has been mostly used for individual- and interpersonal-level interventions, with limited application to organizational level interventions (1–8). In response to these gaps, we intentionally used intervention mapping to develop an organizational level intervention to address the health-harming legal needs (HHLN) of PWH. This article describes our adapted version of intervention mapping to integrate the MLP approach into HIV care. Our learning offers an initial blueprint for communities seeking to strengthen HIV care through developing organizational level interventions to address socio-legal barriers to care—and health—through legal services.

## Background

Since 2018, our investigative team has worked on examining the potential of integrating legal services into HIV care as a mechanism for addressing disparities in HIV care. Medical-legal partnerships (MLP) offer such a potential intervention opportunity. MLP is a healthcare delivery approach that integrates legal services into clinical, behavioral, and social services (9–15). MLPs were developed in order for medical providers to better identify and meet underrepresented patients’ legal needs (16, 17). Though MLPs typically show improved patient outcomes (18), some studies have shown mixed results (19).

From 1993 to 2017, 294 healthcare institutions across 41 states in the U.S. established Medical-Legal Partnership (MLP) programs, offering a multifaceted approach to healthcare delivery by integrating legal advocacy into medicine and health care practices (17, 20, 21). Following the Preferred Reporting Items for Systematic Review, Martinez and colleagues systematically reviewed studies published from January 1993-August 2015 to investigate the capacity of MLP programs to address health disparities and access to justice challenges (13). Thirteen relevant studies from an initial pool of 355 records were identified. Only four studies addressed the effect of MLP interventions on patient health outcomes (22–24, 59). Among these empirical studies, MLPs improved patients' environmental conditions by meeting legal needs and increasing their access to, and retention in, both health services and health-promoting resources such as SNAP (22, 25–27). There also was clear evidence that MLP programs can financially benefit not only patients—through medical debt relief and mitigation—but also the partnering healthcare organizations in the form of reduced organizational costs (24, 28, 29).

MLPs have the potential to improve HIV continuity of care (30–33). Yet MLPs are underutilized in systems of care serving people with HIV or to specifically address their legal needs (34, 35). Implementation science methodologies, such as intervention mapping, can facilitate the adoption and integration of existing intervention packages into health and social services organizations (36–38).

To advance the growing field of implementation science in public health, this manuscript presents a practical application and adaptation of intervention mapping to support the integration of MLPs into HIV care settings. Specifically, we outline a modified five-step intervention mapping process used to design and implement OPAHL (Organizational Partnerships for Healthy Living), an organizational-level intervention aiming to mitigate the impact of negative social determinants of health on persons with HIV. The objectives of this manuscript are to (1) describe the rationale and methodology behind adapting the traditional six-step intervention mapping protocol, (2) detail the steps used to co-develop and tailor MLP implementation strategies in HIV care settings, and (3) present the resulting intervention framework currently being evaluated for feasibility and preliminary effectiveness.

## Methods

### Participants and recruitment

We identified programs with existing or planned services for persons with HIV (PWH) and invited MLP staff (e.g., attorneys, case managers, paralegals) and affiliated HIV care providers (e.g., clinicians, social workers, administrators) to participate. Participants were recruited via email and direct outreach from project collaborators.

## Data collection

To inform Step 1 of intervention mapping, we conducted a mixed-methods implementation needs assessment using both survey instruments and semi-structured interviews. Surveys included both closed- and open-ended questions on current MLP activities, perceived barriers and facilitators to integration with HIV services, training needs, and organizational readiness. Interview questions further explored program structure, collaborative practices, staff roles, communication processes, and observed legal needs among PWH. We also collected relevant documentation from MLPs (e.g., intake forms, training materials, workflows) for supplemental content analysis.

## Thematic analysis

We applied thematic analysis (39) to the open-ended narrative responses from interviews and survey instruments, following Braun and Clarke's six-phase framework: (1) familiarization with data, (2) generating initial codes, (3) searching for themes, (4) reviewing themes, (5) defining and naming themes, and (6) producing the report. Data were coded independently by two members of the research team using both deductive codes derived from constructs and inductive codes that emerged from the data. Discrepancies were resolved through consensus meetings, and coding consistency was assessed through inter-rater reliability checks. Emergent themes were mapped to provide a structured understanding of MLP implementation in HIV settings. In addition to thematic analysis, content analysis of program materials was conducted to identify the most common components and workflows in use across sites. This dual approach enabled us to triangulate findings and identify key implementation barriers and facilitators, which informed the development of logic models, performance objectives, and strategy selection in subsequent intervention mapping steps.

### Application to intervention mapping steps

Empirical evidence exists supporting the use of intervention mapping as an innovative methodological approach for building intervention models that can be implemented and tested (40, 41). The design of sustainable interventions that achieve desired outcomes requires transparent, rigorous processes (5). For these reasons, we used the intervention mapping approach, as opposed to PRECEDE-PROCEED, ADAPT-ITT, colocation or other intervention design methodologies to design our MLP integration into HIV care services intervention package.

**Figure 1:**
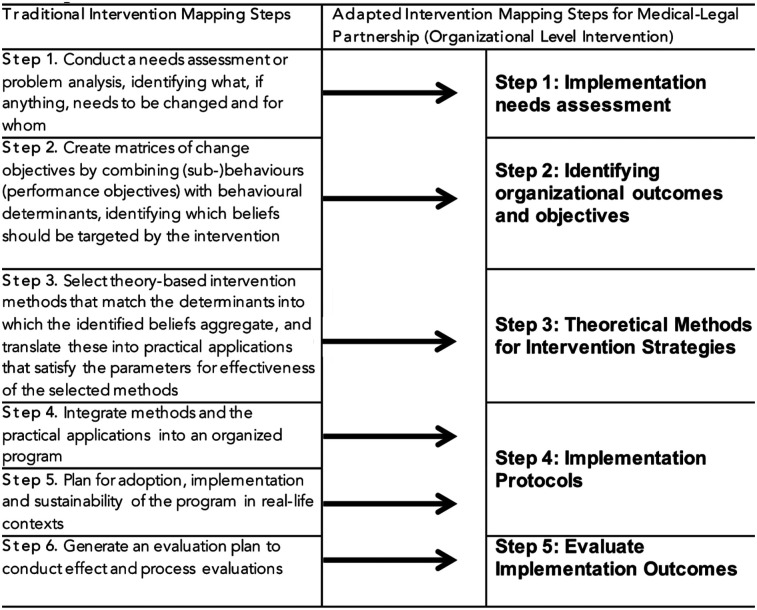
Adapted intervention mapping steps for development of an intervention to integrate a medical-legal partnership approach to HIV care services.

Figure 1 illustrates our adapted five-step intervention mapping process. While we retained the core principles of the original intervention mapping framework, our most significant methodological adaptation was the integration of Step 4 (program production) into Step 5 (adoption and implementation). Step 4 traditionally focuses on organizing intervention components into a coherent program, but through our initial needs assessment (Step 1), we identified that robust, independent intervention packages already exist for both legal services (via Medical-Legal Partnerships) and HIV care. These interventions are implemented by trained professionals in their respective domains—attorneys and medical providers—following established protocols. Rather than reconfigure these interventions at the patient level, our analysis revealed the greater opportunity and need to integrate them at the organizational level while maintaining their separate identities during patient service delivery. This strategic decision ensures that patients benefit from co-located but distinct services that preserve professional boundaries while fostering collaborative care.

This shift in focus required an enhanced emphasis on the systems-level integration, implementation logistics, and sustainability planning typically housed in Step 5. We prioritized understanding how to embed MLPs within existing HIV care infrastructure in ways that are feasible, acceptable, and effective. This aligns with the flexibility endorsed by the developers of intervention mapping, who recognize that certain steps may be modified or omitted based on the intervention context and readiness. As such, our adapted five-step model includes: (1) conducting an implementation needs assessment, (2) identifying multi-level outcomes and objectives, (3) selecting theoretical methods and change strategies, (4) developing site-specific implementation protocols, and (5) evaluating implementation outcomes. This tailored approach allowed us to create an integrated yet scalable intervention grounded in real-world conditions and informed by community and provider perspectives.

## Results

The first step of our intervention design was to convene a planning advisory working group consisting of two collaborative boards (scientific and community), drawing on Pinto et al. (42) approaches to community engagement in HIV research. The community collaborative board met in person at Temple University College of Public Health with an attendance of 30 individuals in that initial meeting. The scientific advisory board first meeting was online to discuss the project. From the community and scientific collaborative board we created a subgroup, the planning advisory working group which included: (1) investigators, (2) a core group of eight MLP practitioners who are experts in their fields, including clinical care, legal services, health care administration, behavioural and social services (3) four health services researchers with expertise in the HIV care continuum; and (4) twenty people with HIV, adults, residents of Philadelphia, Pennsylvania. We organized ten planning advisory working group meetings around the intervention mapping steps.

### Adapted intervention mapping step 1: implementation needs and assets assessment

The planning advisory group provided feedback to the investigative team in designing the needs and assets assessment. We decided to conduct and need and assets assessment that consisted of three components: (1) online survey with existing MLP practices that may serve PWH; (2) online survey with PWH who have received legal services to address their HHLN; (3) open-ended interviews with MLP practitioners; and (4) content analysis of secondary information provided by MLPs. The list of MLPs was obtained from the National Center of Medical Legal Partnerships. To participate in the assessment, individuals had to verify that (1) they were part of an operational MLP program with integrated legal services, on-site or off-site; (2) the program had been operating for at least one year and they had been part of the program's service delivery for at least one year; and (3) at least fifty percent of the patients served by the program, during the six months prior to our research communication, were PWH.

The objectives of the needs/assets assessment were: (1) to identify existing best practices among current MLP programs to address legal barriers to care and health for clients/patients with HIV; and (2) to assess the effects of current MLP programming and practices on HIV care continuum indicators. The online survey and open-ended interview instruments were designed using the EPIS framework for examining inner and outer factors in adopting innovations (43). In this case, the potential innovation is the integration through colocation of legal services in HIV services facilities. Recruitment for the online survey was a challenge, with close to 20% response from all MLPs nationwide, and 15% meeting our organizational eligibility criteria. A subsample of the survey participants was selected to participate in the open-ended interviews. Selection was based on availability and willingness to participate in the interviews. Open-ended, informal interviews were conducted in person with 6 providers at The Philadelphia AIDS Consortium (Philadelphia, PA- 1 participant), Betances (New York, NY-1 participant), Us Helping Us (Washington, DC-1 participant), and Whitman Walker (Washington, DC-3 participants) Further, in Step 1 we were able to recruit *n* = 111 MLP practitioners who participated in the online survey with existing MLP practices that may serve PWH, including providers from for-profit (0.9%) and non-profit organizations (62.2%) as well as some unknown (36.9%). Providers who completed the surveys were Administrators (15.3%), Clinicians (22.5%), Lawyers (36%), and Social Service providers (26.1%) (44).

The investigative team conducted tabulated the online survey findings and conducted basic descriptive statistics. We also conducted thematic analysis of the open-ended interview narrative materials and content analysis of the reports and materials provided by the MLPs to determine the common themes in barriers and facilitators to addressing HHLN for PWH.

A majority of providers (76.9%) identified the need for better tools to screen for HHLN, while 39.1% reported limited collaboration with legal partners. Organizational capacity was a major concern, with one administrator stating, “*We have only been operating for 2 years, and we are a staff of two—one MLP attorney and one program coordinator. We could already use another attorney*”. Structural limitations were also noted: “*Coordinating the logistics of all the services we provide with limited staff*” (social/behavioral health provider), and “*Funding. Funding. Funding*”. (lawyer) were common refrains. Legal providers also highlighted the burden of being under-resourced, especially in time-intensive cases: “*Legal cases take time and one lawyer cannot handle as many cases as one doctor sees patients*”. Systemic misalignment between healthcare and legal domains was another theme: “*Medical providers’ policies aren’t always clear… we get sent back and forth between supervisors*”, and “*Hospital partners don’t always respect attorney-client confidentiality or how time sensitive fixing problems can be*” (lawyers). These concerns reflected deeper interprofessional tensions and challenges in establishing effective interdisciplinary workflows.

Despite these barriers, the assessment also documented important facilitators and benefits. Providers praised the model's ability to reach clients who otherwise would not seek legal help: “*One-stop shop for patients who need services. we feel we are able to address a population who does not actively seek out legal aid*” (lawyer). Integration into clinical workflows enabled more holistic care: “*It's so nice to work as a team with social workers and providers to give the best service to patients*” and “*Working in a health setting and in collaboration with a healthcare provider improves patient care*” (lawyers). Several lawyers emphasized the health impact of legal services: “*The help of a lawyer can improve patient health*”, and “*An MLP is able to identify and address root legal issues before they reach a crisis point and cause health crises*”.

Thematic findings from this assessment were triangulated with survey results and presented to the planning advisory group, which used them to inform two logic models: (1) a logic model of risk focused on unaddressed HHLN for PWH, and (2) a logic model of change outlining necessary organizational and system-level shifts to detect and mitigate HHLN. From these, we established our primary health outcome: reducing HIV viral load to undetectable levels within six months of care engagement through the resolution of legal and social barriers.

Five key stakeholder groups were identified to operationalize this intervention: (1) PWH with unsuppressed viral loads and HHLN; (2) healthcare and social service providers in community health centers; (3) administrators and organizational leaders; (4) legal aid organizations geographically proximal to HIV service sites; and (5) attorneys providing direct legal services.

### Adapted intervention mapping step 2: identifying stakeholders' outcomes and performance objectives

In this step 2, we develop matrices that combine the desired behaviors identified in the logic model of change and the hypothesized environmental conditions, factors, and determinants that lead to those desired behaviors, thus creating measurable competencies (defined here as objectives that will result in the desired change). Below we list the desired behaviors by type of stakeholder:

• *Administrators of partnering community health centers and legal aid organizations*: (1) facilitating the implementation of MLP programming meetings with internal operations and external collaborators; and (2) providing ongoing support for the effectiveness and sustainability of the MLP program.
• *Medical, health, and social services providers*: (1) identifying HHLN within clinical, behavioural, and social services encounters; (2) screening and referring patients with identified HHLN to MLP legal services providers; (3) alerting legal services colleagues to any health conditions that may impact efforts to address HHLN (assuming patients have authorizing this transmission of protected health information); and (4) coordinating communications between medical and legal providers as part of ongoing, sound case management for PWH.
• *Legal services providers*: (1) managing the consent/authorization process with legal clients that can enable the legal team to transmit case update information to the patient's medical and/or social services teams, as appropriate and necessary; (2) reinforcing, as part of that consent/authorization process, that a patient/client still can receive legal services even if they decline to permit communication between legal advocates and medical or social services staff, without any negative impact on the attorney-client relationship (although the lack of information-sharing sometimes can impact the strength of advocacy in certain contexts); (3) confirming the specific legal needs as to which the attorney will provide legal representation to the client; (4) confirming the specific legal needs for which the patient/client will be offered referral to other organizations with aligned expertise and capacity; and (5) providing regular workshops for patients and collaborating medical sites on recurring legal concerns.

In our step 2, we used the matrices to operationalize each of the above desired behaviors further into sub-tasks, or performance objectives. After each performance objective was identified, the investigative team, conducted a literature review of behavioral change among providers and consulted with the planning advisory working group to identify determinants of change, i.e., what cognitive resources, material resources, and affective resources would be needed to motivate the stakeholder to achieve the task. We selected determinants from the Diffusion of Innovations Theory and from Organizational Innovations Change Theory (45–48) and identified six core determinants with various degrees of applicability to each of the performance objectives: (1) awareness knowledge; (2) procedural knowledge; (3) motivation for innovation; (4) outcomes expectations; (5) organizational culture; and (6) reinforcements. Following, Fernandez, et al. (49), we asked what members of the planning advisory group what resources were needed to change in the determinants listed above to accomplish the performance objective. Those responses became the competencies guiding the intervention that was being developed.

#### Conceptual basis of intervention

**Figure 2:**
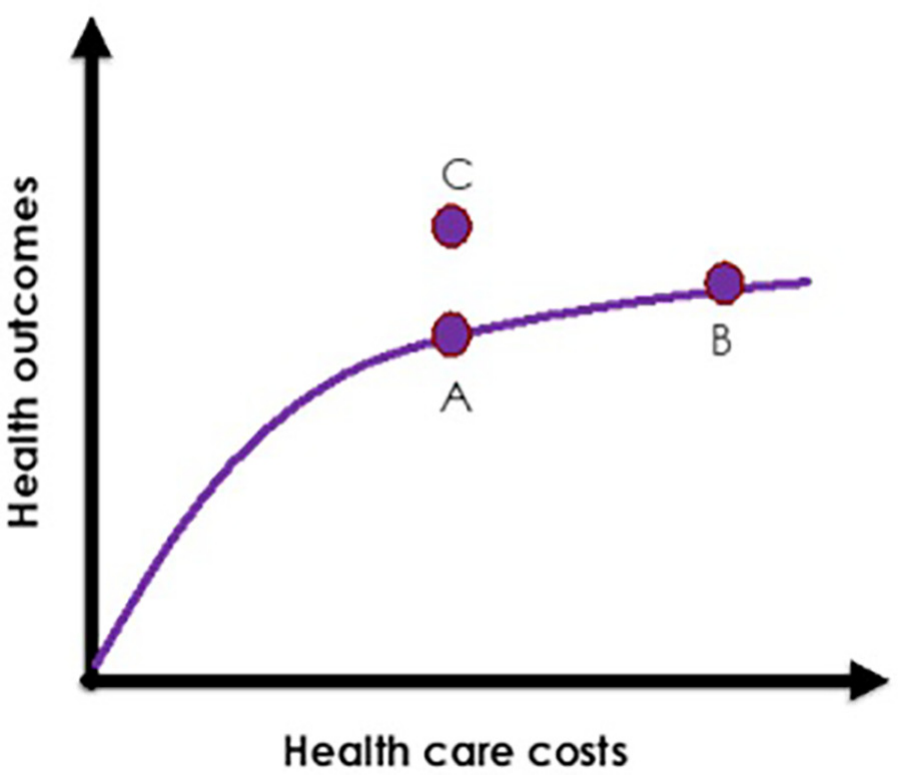
OPAHL synergistic continua of care for people with HIV (PWH).

As a result of Step 1 and 2 exercises and discussions with the planning advisory group, the investigative team developed the conceptual basis of the intervention. Building on the literature of integrative care (50, 51), the conceptual basis of our proposed intervention is two folds: (1) in order to provide comprehensive HIV care, we first must identify the HHLN that reduce or prevent access to care in the first place; and, (2) integration of legal and HIV services should be grounded in a basic economic model of integration and co-location of healthcare services. Thus, we selected the Evans Health Economic model illustrated in Figure 2 to achieve better health outcomes without a significant cost increase (52). In Figure 2, Point B represents higher health care costs for slightly better health outcomes (point A). Point C represents achievement of a better health outcome (than point A) without increased costs (such as in Point B). This conceptual basis was based on discussions of the planning advisory group and would need to be tested empirically to be further validated. The planning advisory group agreed that an MLP PWH who have undetectable viral loads cannot transmit HIV, therefore, increasing the number of PWH who are virally suppressed is a national priority (53). For every person not infected with HIV, there is a lifetime cost savings of $367,134 per person in potential lifetime HIV treatment costs (54).

### Adapted intervention mapping step 3: theoretical methods for intervention strategies

We grouped the competencies created in Step 2 according to their respective determinants. We brainstormed and conducted additional literature reviews of the most effective learning constructs and concrete strategies to achieve the designated competencies. For example, in Step 2 we identified the key competencies for medical, health and social services providers to achieve both individual- and organization-level impacts: (1) Explain the importance of MLP services for PWH; (2) Explain the MLP program structure for PWH; (3) List the problem-solving strategies to connect and participate in MLP from the perspectives of providers and patients; (4) Express positive disposition towards innovative, holistic services for PWH; (5) Express positive disposition towards screening for health-harming legal needs; (6) Express support for integration of legal services into HIV care; and (7) Understand that MLP program implementation will reduce health care costs for out of care PWH. This intervention should increase awareness, knowledge, motivation for innovation, and outcome expectations (briefly defined as subjective estimates of how likely it is that a specific behaviour will be followed by consequences) through the following educational methods: (1) consciousness raising, (2) chunking of information, (3) information transfer, (4) persuasive communication, and (5) constructive argument.

Strategies for effective implementation included tailoring of best-practice communication and information- sharing protocols, anchored in patient autonomy and choice. Through this design step, we decided on using the following educational-intervention strategies by: (1) person-to-person meetings with administrators of health centers and legal aid organizations; (2) in-person training on HHLN for medical, health and social services providers; (3) in-person training on the HIV continuum of care for legal services providers.

**Figure 3:**
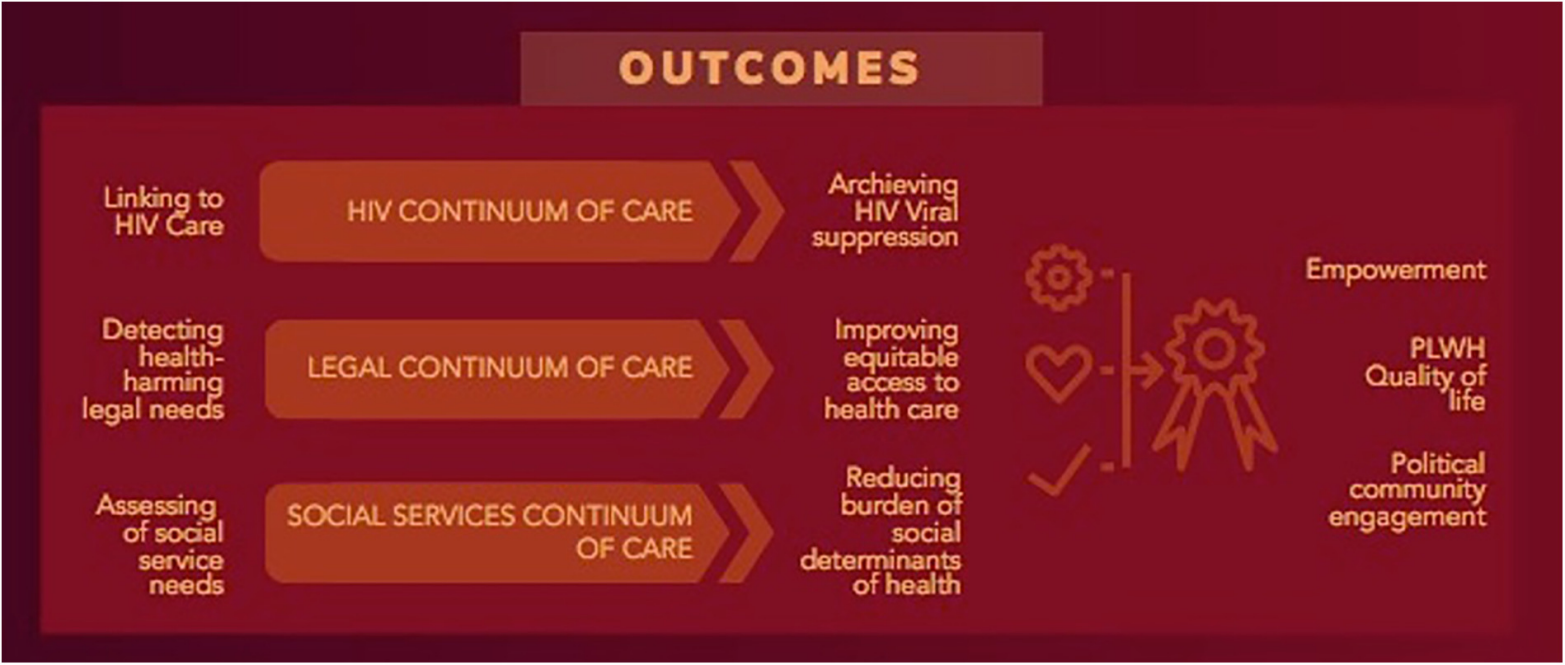
Illustration of Evan's Health Economic model.

At the end of this Step, we collected all the educational strategies together and identified five stages for education of stakeholders: identification of health-harming legal need(s); offering of, and referral to, legal services; engagement in legal services; resolution of legal concern(s); and ongoing access to legal support (see Figure 3).

### Adapted intervention mapping step 4: implementation protocols

This step focuses on building a plan for building and implementing the intervention.

#### Generating the name of the intervention

A member of the team suggested the name, *Organizational Partnerships for Healthy Living*, OPAHL, because of the importance of building interorganizational partnerships. The name was adopted for our MLP intervention package.

#### Development of program materials

In Steps 2 and 3, we developed and cross-checked all the content for each component of the intervention. Because of the high level of prior training and education of our intervention targets (e.g., licensed attorneys, licensed physicians and nurses) at the organizational level, all materials geared to organization-level impact were developed at a high level of literacy. Lawyers, physicians, psychologists, health educators, and social workers of the advisory planning group were asked to provide feedback in the review of intervention materials to identify and simplify professional jargon.

#### Program overview

Our intervention leverages medical care expertise to enable access to legal support, potentially increasing the benefits to detecting and addressing HIV patients experiencing HHLN. Three factors were identified by the advisory planning group as necessary for a feasible MLP program implementation: (1) coordination of clinical, behavioural, social, and legal services; (2) an effective protocol to identify HHLN and offer/refer patients for legal support; and (3) siting of legal services on-site at the health clinic. Against this backdrop, the OPAHL intervention package consists of three components.

***Component 1*** is a comprehensive training for all MLP care providers delivered to clinical, behavioural, social, and legal staff together to establish a collaborative environment and relationship. This training is to be delivered by an expert team experienced in MLP implementation.

***Component 2*** consists of the screening tool and screening protocol to identify health-harming legal needs of PWH patients. The screening tool, based on a screening tool developed by the National Center for Medical-Legal Partnership, provides a comprehensive guide to identify potential legal concerns of PWH patients (55). This screening, administered by a member of the clinic's staff, offers the initial point of entry to potential legal services. This is a core node of the OPAHL intervention, as a positive screening (a screening is positive if at least one legal need is identified) triggers the initial offer of connection to legal support.

***Component 3*** includes the provision of legal services by legal advocates. Upon a positive legal needs screening, a case manager or social services provider connects the patient to the co-located lawyer, giving the patient the option to engage the services of the attorney and address the identified legal concern(s) with legal representation.

The OPAHL training curriculum is a combination of readings and in-person training that provides the knowledge and skills needed to integrate legal and HIV services. The content covers a variety of topics, including social determinants of health; HHLN and their impact on PWH; legal aid as health care; HIV synergistic continua of care; and MLP in action. The training is designed to be conducted in collaboration with organizations and/or experts experienced in MLP implementation.

We piloted the OPAHL training manual in a two day in-person sessions with members of an HIV services and a legal aid organization in Philadelphia. We modified the training based on the sessions feedback resulting in three pre-training readings (approximately 2 h) slide-animated presentations (approximately 1 h), and two-day sessions of interactive discussions delivered in-person (approximately 7 h, exclusive of breaks). The OPAHL training focus is not on the practices of law, medicine, nursing or social work, but rather how systems thinking with regards to the detection of HHLN and the linkage and retention in legal care.

In addition to the implementation characteristics noted above, this Step of the intervention mapping process helped to clarify important features of the scope and limitations of OPAHL. OPAHL will offer direct legal services for a broad range of civil law (as opposed to criminal law-based) needs that impact health. Most legal aid organizations are funded by the Legal Services Corporation (LSC), a publicly funded non-profit corporation established by the U.S. Congress in 1974. LSC funding prohibits the use of its funds for representation of people in several contexts, including criminal cases and several categories of people who are not U.S. citizens; limited exceptions exist. Thus, OPAHL likely will not directly enhance patient access to criminal law defense resources; and depending on the funding of the legal partner characteristics, OPAHL may bolster access to immigration law resources for PWH.

#### Ideal site for implementation

The planning advisory group decided that to most effectively monitor the implementation of OPAHL in achieving its health outcomes, it is best to be implemented in federally qualified health centers (FQHC). FQHC have the infrastructure and capacity of maintaining health and social services records for patients; provide on-site medical care; and conduct a comprehensive evaluation of the OPAHL implementation (see Step 5).

#### Planning for sustainability

Potential participating FQHCs will sign letters of support. The OPAHL planning advisory group designed a “pitch” one-to-one intervention activity with administrators consisting of three in-person meetings with the OPAHL team and the leadership of at least one local legal aid organization. As part of this step, the intervention team works with the selected FQHC and legal aid collaborators to create a Memorandum of Understanding (MOU). This MOU outlines the mission and goals of the partnership(s), designates roles and responsibilities between FQHC and legal services, and identify the organizational contributions of all partner organizations. The initial expectation is that the FQHC will provide legal colleagues with office space, computer, phone, and necessary access to patient files and records. The legal aid organization(s), in coordination with the intervention team, will minimally designate protected time of an attorney licensed to practice in the state as well as protected time of paralegal resources, and will provide to that legal team office space as well as telephones and computer systems at the legal aid office. Within 4–6 months of executing the Memorandum of Understanding, it is expected that the components described above under Step 3 will begin.

Two tasks are critical for the adoption-implementation step: (1) the internal collection of data on exposure to the intervention components, and (2) the monitoring of patients'/clients' progress along the HIV continuum of care and the phases of MLP-enabled legal advocacy. For the first task, OPAHL requires the OPAHL staff paralegal, in collaboration with FQHC staff, to collect health and social services exposure data, following an OPAHL- designated protocol (assuming patients have consented to this information transmission). For the second task, attorneys will coordinate with FQHC administrators to integrate de-identified MLP program observations and trends into regular clinic dialogues.

### Adapted intervention mapping step 5: evaluate implementation outcomes

Guided by the adapted intervention mapping steps 1 through 4, we specified the desired intervention outcomes, predictors, mediating variables, and process evaluation indicators (see Table 1). The intervention mapping process also enabled us to identify the most appropriate evaluation models. We utilized the RE-AIM (Reach, Effectiveness, Adoption, Implementation, and Maintenance) and the EPIS frameworks to design an evaluation plan. In this Step, we develop a feasibility trial proposal to examine the implementation and preliminary efficacy of OPAHL. Together with the planning advisory group, and other collaborative organizations, we were externally funded to evaluate OPAHL in Philadelphia, PA. In Step 5, we added additional members to the planning advisory group, including epidemiologists, health economists and statisticians. Completing Step 5 of intervention mapping was critical in developing an evaluation plan that was realistic and feasible to be implemented in three-way organizational partnership: health care center (FQHC), legal aid services organization, and investigative-research organization.

**Table 1:** Implementation indicators guided by Re-AIM (56).

Objectives	Measures (Indicator of success)
(1) To assess the feasibility of the processes that are critical to the success of the subsequent efficacy trial.	1. Recruitment rates (>10 individuals per week) 2. One-month retention rate (>80%) 3. Refusal rate after describing intervention (<50%) 4. Adherence rate to OPAHL intervention protocol (>75%) 5. Eligibility criteria (# people excluded & reasons) 6. Understanding of self-assessment tools (>95%; # of unanticipated answers to study questions)
(2) To identify the time and resource problems that can occur during the subsequent trial.	1. 1. Length of time to fill out all the study forms (<20 min) 2. Impact of participants to overall flow of patients in the organization (Negligible) 3. Length of time to complete baseline assessment (<60 min) 4. Level of missing data (<5%) 5. Space for OPAHL facilitation (availability) 6. Unexpected needs for resources (office space, transportation, meals) 7. Levels of data errors in entry/management (<5%) 8. Adequacy of data systems to capture, store and analyze data (>95% compliance) 9. Implementation time (Implementers perform the tasks within the timeframe) 10. Evaluation time (Investigators perform the tasks within the timeframe)
(3) To forecast potential management problems.	1. Communication challenges between implementers and investigators 2. Minimum level of training necessary to implement intervention

## Discussion

Epidemics driven by structural factors, such as HIV, require multi-level interventions that account for individual, organizational, and structural variables. Intervention mapping allowed the planners, through iterative processes, to identify key domains of intervention, leverage important assets, and design a multilevel, evidence-based intervention. Intervention mapping facilitates planning and design for dissemination, implementation, and maintenance of evidence-based interventions in practice. Given the promise of ecological approaches, we implemented intervention mapping methodology identified by Fernandez, et al. (5, 36) including the application of theory, evidence, and incorporation of care and service providers and community stakeholders into the intervention design process (36).

We identified four challenges in the process of designing OPAHL using intervention mapping. First, most intervention design methodologies are geared to individual-level (patient-level) interventions and outcomes. To overcome this challenge, we drew heavily on the qualitative data collected during Step 1 of our adapted intervention mapping process. This qualitative data reflected important inputs from MLP program delivery actors (largely professionals and paraprofessionals) who could offer observations of how their organizations' operational and cultural characteristics can impact successful MLP program implementation, impact, and sustainability. Second, there are inherent epistemological differences between the fields of medicine, behavioral health, social services, law and public heath that can affect their frameworks for intervention planning. However, integration of services aligned with each of these fields (and likely additional fields) is essential to tackle the growing HIV epidemic in the U.S.; particularly among subgroups including immigrants, sexual and gender minorities, and racial/ethnic minorities. Preliminary research has documented the positive impacts of care integration approaches, including legal services integration, on individual-level health outcomes (11, 57). Third, the study findings revealed that diverse systems of care deploy a range of documentation systems (e.g., electronic health records, legal case management databases, mental health records that are subject to a higher level of confidentiality and privacy, etc.) and are subject to a range of professional responsibility obligations relating to confidentiality and mandated reporting of suspected neglect or abuse. However, recent literature has shed light on potential strategies to address confidentiality navigation and management challenges (58), including the creation of comprehensive data-sharing standard agreements (for organizations) and informed consent forms (for patients) that clearly explain allowable data usage and access. Fourth, in the healthcare ecosystem, which occupied 17.3% of U.S. Gross Domestic Product in 2022, there is profound concern about the economic sustainability of integrated approaches to care; especially those perceived to require large, ongoing funding streams to be sustainable. We urge researchers to promote the importance of multi-level, sequenced implementation steps to iteratively design for sustainability. In addition, research studies should be conducted to assess the cost-effectiveness of MLP program interventions.

Using intervention mapping was time consuming. The time and effort dedicated to assembling, organizing, and facilitating working meetings with the planning advisory working group was significant, but relatively negligible compared to the time and effort expended “behind the scenes” preparing materials for each meeting and completing deliverables stipulated by the planning advisory working group. We completed Step 1 in 12 months. A total of 616 salaried hours of intervention designers' time, and 1,400 salaried hours of staff time, were dedicated to Step 1. We completed Steps 2 through 5 in approximately 6 months, with an estimated total of 403 salaried hours of salaried intervention designers' time, and 840 salaried hours of staff time were dedicated to these steps. We found the use of intervention mapping critical to supporting design rigor and intervention quality for the OPAHL package; this presents a major advantage in designing a multi-level intervention that has high potential for adoption and sustainability. After the development of the OPAHL intervention package, OPAHL was funded by the National Institute of Mental Health (NIMH) to be tested for large scale feasibility and preliminary effects in randomized cluster trial in the city of Philadelphia, Pennsylvania, United States (grant # 1R34 MH125718-01A1; 2021–2024).

An important implication of this work lies in its alignment with the shifting priorities of the National Institutes of Health (NIH), particularly amid recent funding cuts to HIV prevention and care. Rather than retreating from structural interventions, federal agencies should expand investment in community-informed, multi-level strategies that address the root causes of health inequities. Our findings offer a timely, evidence-based framework for integrating MLPs into HIV service delivery. Developed through a rigorous, participatory process, the OPAHL intervention exemplifies a sustainable and scalable approach that embeds social determinants of health into care models. This work underscores the urgent need for stable, long-term funding mechanisms that support complex, structural interventions beyond short-term demonstration projects.

### Limitations

This study has several limitations. Firstly, the modification of the intervention mapping process from a six-step to a five-step approach may have led to oversimplification or omission of critical components, potentially impacting the comprehensiveness and effectiveness of the intervention design. Additionally, the needs assessments conducted among Medical-Legal Partnership (MLP) programs may not fully capture the diverse perspectives and experiences of all stakeholders involved, including providers, patients, and community members. This could introduce biases or overlook important contextual factors that influence the implementation of comprehensive legal services in HIV care settings. Furthermore, while intervention mapping emphasizes iterative processes and stakeholder engagement, the extent to which these principles were effectively implemented in our study may vary, potentially impacting the fidelity and sustainability of the intervention. Lastly, the feasibility and preliminary effectiveness testing of the intervention package, OPAHL, in a hybrid, randomized cluster trial in Philadelphia, Pennsylvania, may not fully generalize to other settings or populations, limiting the external validity of our findings. These limitations underscore the need for further research and refinement of intervention mapping methodologies to address complex public health challenges comprehensively and effectively.

## Conclusion

Adapting intervention mapping enabled the development of a multi-level MLP intervention package with the potential to strengthen HIV care continuum outcomes by addressing health-harming legal needs and underlying social determinants of health. Aligning legal services with emerging patient needs is essential to advancing national HIV treatment priorities. This study provides a replicable framework for researchers and practitioners designing structural interventions in contexts where care access and treatment engagement are shaped by complex social and systemic factors—particularly in chronic and communicable disease settings like HIV. Importantly, this work is especially relevant amid recent shifts in NIH priorities and reductions in federal HIV funding. Rather than moving away from structural approaches, sustained investment in scalable, community-informed solutions like OPAHL is urgently needed. Our findings highlight the importance of long-term funding mechanisms that support real-world implementation of equity-driven, multi-sector interventions.

## Data Availability

The datasets presented in this article are not readily available due to the nature and sensitivity of the qualitative data presented. Requests to access the datasets should be directed to the corresponding author.
